# Supplementary material for: A toll-like receptor agonist mimicking microbial signal to generate tumor-suppressive macrophages
Source: Nat Commun. 2019 May 22;10:2272. doi: 10.1038/s41467-019-10354-2 (PMC6531447; doi:10.1038/s41467-019-10354-2)
Supplement: Supplementary file 1 — Supplementary Information [file 41467_2019_10354_MOESM1_ESM.pdf]

Supplementary Information

A toll-like receptor agonist mimicking microbial signal to generate tumor-suppressive macrophages

Feng et al.

A toll-like receptor agonist mimicking microbial signal to generate tumor-suppressive macrophages

Yanxian Feng[1], Ruoyu Mu[1], Zhenzhen Wang[2], Panfei Xing[1], Junfeng Zhang[2], Lei Dong[2], and Chunming Wang[1]

[1] State Key Laboratory of Quality Research in Chinese Medicine, Institute of Chinese Medical Sciences, University of Macau, Macau SAR

[2] State Key Laboratory of Pharmaceutical Biotechnology, Nanjing University Institution, Nanjing, 210093, China

[#] These authors contributed equally: Yanxian Feng, Ruoyu Mu.

Correspondence and requests for materials should be addressed to: C. Wang: cmwang@umac.mo; or L. Dong: leidong@nju.edu.cn

Supplementary Methods

Reagents. All the chemicals used in this study were purchased from Sigma-Aldrich (USA) unless otherwise stated below. Konjac glucomannan (KGM, Mw 100,000) was provided by Shimizu Chemical (Japan). The glucomannan consists of β-1,4 linked D-mannose and D-glucose monomers with a molar ratio of around 1.6:1 (mannose to glucose). Its quality was compared against numerous other commercially available samples and ensured by preliminary processing and characterization, part of which has been demonstrated in our previous studies.[1] Trifluoroacetic anhydride (TFAA), pyridine, acetic anhydride N, N-dimethylformamide (DMF), 1, 8-diazabicyo [5, 4, 0]-7-undecene (DBU), fluorescein-5-thiosenicarbazide (FITC), and monodansylcadaverine (MDC) were purchased from Aladdin (China). Pam_3CSK_4, and Pam_2CSK_4 were purchased from Invivogen (France), Protein G PLUS-Agarose was purchased from Santa Cruz Biotechnology (USA). Clodronate liposomes were purchased from Liposoma Technology (Netherlands).

Isolation of peritoneal macrophages. Mice were sacrificed and intraperitoneally injected with 8 mL of PBS and massaged for 1 min before pumping back. The peritoneal macrophages were cultured in RPMI 1640 cell culture medium containing 10% (V/V) FBS (Gemini Bio Products) at 37 °C.

Isolation of bone marrow derived macrophages (BMDM). Total cells were harvested by flushing cells from femurs and tibiae of mice and cultured on non-adherent petri dish with cell culture medium containing 10% (V/V) conditioned medium from L929 cells. After 7 days incubation, BMDM were collected after cell culture medium was changed and the unattached cells were removed. The phenotype and purity of these primary macrophages were routinely checked in our laboratories.

BMDM were stimulated into M1-phenotype with LPS (100 ng/mL) and IFN-γ (40 ng/mL) treatment for 48 h, or into M2-phenotype with IL-4 (40 ng/mL) and IL-13 (20 ng/mL) treatment for 48 h. Successful M1/M2 polarization was verified by flow cytometry and RT-qPCR.

The reporter cells were HEK293 cells co-transfected with murine TLR2 or TLR4 and SEAP (secreted embryonic alkaline phosphatase), and the level of SEAP could be conveniently detected by adding HEK-Blue Detection agent and reading the absorbance at 650 nm.

Endocytosis assay. To prepare FITC-labeled particles, acGM-0.2 or acGM-1.8 (100 mg) was mixed with FITC (10 mg) in a mixture of DMSO-HOAC (7:3, V/V, 10 mL). The mixture was stirred at room temperature for 30 min, followed by addition of $NaBH_4$ (500 μL, 0.4 M) and incubation at 70 °C for 2 h. The products were purified by dialysis (avoiding light) and lyophilized.

BMDM were seeded on cover glasses, growing for 1 day, pretreated in the presence or absence of monodansylcadaverine (MDC, 100 μg/mL, 30 min), and incubated with FITC-labeled acGM (100 μg/mL, 2 h). Nuclei were counter-stained with DAPI (100 ng/mL, 10 min). After incubation, the cells were rinsed with PBS, fixed in paraformaldehyde (4%, 10 min), and rinsed again with PBS for three times. All images were captured by a TCS SP8 confocal laser scanning microscope (Leica Microsystems, Heidelberg, Germany).

In parallel, after incubation with FITC-labeled acGM or GM, the supernatant was collected, and related cytokines were determined by ELISA. The cells were scraped off, collected by centrifugation (1000 rpm, 5 min), washed with ice-cold PBS for three times, and tested by BD Accuri C6 flow cytometry.

***In vitro* anti-tumor assessment of acGM-1.8-challenged macrophages.** BMDM were co-incubated with acGM-0.2 or acGM-1.8 (25, 50 or 100 μg/mL). After 24 h, the culture medium was collected. S180 sarcoma or B16-F10 melanoma cells were seeded on 96-wells culture plate (5×10^3 per well) and incubated overnight, before culture medium was replaced by the collected macrophage medium. After 48 h, cell viability was determined by CCK-8 assay. In parallel, acGM-0.2 or acGM-1.8 was directly added to the culture medium of the tumor cells.

Flow cytometry assay. All samples were stained with antibodies or isotype control. All samples were gated on FSC-A/SSC-A to remove debris. FITC-A, PE-A, APC-A and Cyc5.5-A were used to differentiate positive or negative populations. Cells stained with isotype control was used to define the negative population. Gating strategies were illustrated in Supplementary Figure 15.

ELISA. For *in vitro* samples, cell culture was collected and centrifuged, and the supernatant (100 uL) was added to each well of 96 wells-plate for ELISA testing; for tumor tissue, after weight normalization, the tissue was homogenized in PBS and centrifuged to collect the supernatant for the determination of related cytokines. All ELISA experiment was performed in triplicate and repeated for three times.

Quantification of immunofluorescent images. To quantify the specific cytokine/marker expression, we randomly selected five zones (in the same size) per image from three images and quantified the intensity of the specific signal and DAPI.

Supplementary Table 1. Primer sequences used for RT-qPCR.

Cytokines	Forward	Reverse
**TNF-α**	ACGGCATGGATCTCAAAGAC	AGATAGCAAATCGGCTGACG
**IL-1β**	GCAACTGTTCCTGAACTCAACT	ATCTTTTGGGGTCCGTCAACT
**IL-12 p40**	AGCAGTAGCAGTTCCCCTGA	AGTCCC TTTGGTCCAGTGTG
**IL-10**	GCTCTTACTGACTGGCATGAG	CGCAGCTCTAGGAGCATGTG
**TGF-β1**	TGGAGCAACATGTGGAACTC	TGCCGTACAACTCCAGTGAC
**Arg-1**	CAGAAGAATGGAAGAGTCAG	CAGATATGCAGGGAGTCACC
**GADPH**	AACGACCCCTTCATTGAC	TCCACGACATACTCAGCAC

Supplementary Table 2. The antibodies used in this study.

Application	Antibody	Catalogue No.	Manufacturer	dilutions
**Flow cytometry (anti-mouse)**	FITC-F4/80	123107	Biolegend	1:100
	PE-CD11c	557401	Biosciences	1:100
	APC-CD11b	101212	Biolegend	1:100
	APC-CD206	141707	Biolegend	1:100
	APC-CD163	17-1639-41	eBioscience	1:100
	PE-CD45	103105	Biolegend	1:100
	Cyc5.5-CD45	103131	Biolegend	1:100
	APC-CD3	100235	Biolegend	1:100
	FITC-CD4	100510	Biolegend	1:100
	APC-CD8a	100712	Biolegend	1:100
	PE-CD8a	100707	Biolegend	1:100
	PE-FoxP3	126403	Biolegend	1:100
	APC-Ly6G, 1A8 clone	127613	Biolegend	1:100
	PercP-eFluor-CD170	46-1702-82	Invitrogen	1:100
**Immunofluorescence (anti-mouse)**	CD11c	ab11029	abcam	1:100
	CD206	ab64693	abcam	1:100
	Ly6C	ab15686	abcam	1:100
	IL-10	ab9969	abcam	1:100
	VEGF-A	ab51745	abcam	1:100

	IFN-ϒ	133566	abcam	1:100
**Western blotting**	TLR2	ab24192	abcam	1:1000
**Neutralization**	Anti-mTLR2-IgG	mabg-mtlr2	Inviogen	
	Anti-TLR1 antibody	mabg-htlr1	Inviogen	
	Anti-TLR6 antibody	mabg-htlr6	Inviogen	

a

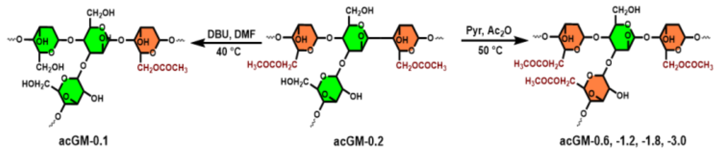

b

Reaction	Time (h)	Acetyl group (%)	DS	Sample name
Deacetylation	2	2.97	0.12	**acGM-0.1**
Starting GM	0	5.98	0.24	**acGM-0.2**
Acetylation	12	14.41	0.63	**acGM-0.6**
	24	19.85	1.23	**acGM-1.2**
	48	24.04	1.81	**acGM-1.8**
	72	45.00	3.02	**acGM-3.0**

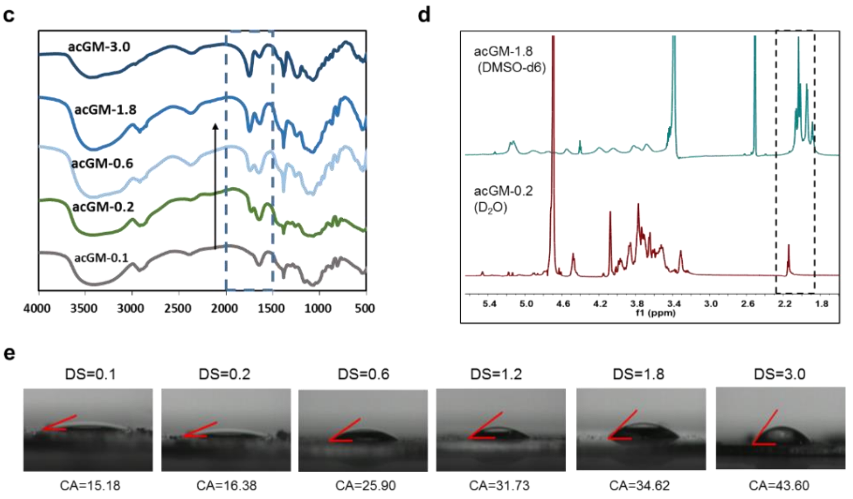

Supplementary Figure 1. Preparation and characterization of acetyl glucomannan (acGM) samples. (a) Schematic illustration of preparing acGM (starting at -0.2) with increasing (-0.6, -1.2, -1.8, -3.0) or decreasing (-0.1) acetylation degrees. (b) Reaction time, acetyl degrees and the names of different acGM samples. (c) Characterization of acGM with different acetyl degrees by infrared spectroscopy. (d) Characterization of acGM-1.8 and acGM-0.2 by ^{1}H nuclear magnetic resonance (^{1}H NMR). (e) Measurement of the contact angle of acGM with different acetyl degrees. acGM was dissolved in $CHCl_3$ and dropped on glass surface; after the solvent was evaporated, the contact angles were measured.

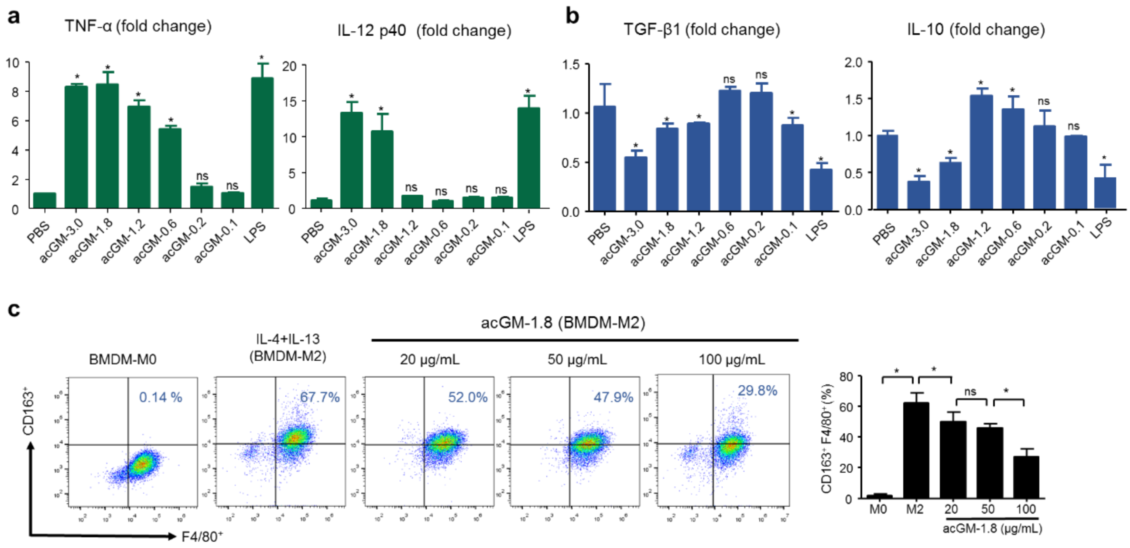

Supplementary Figure 2. acGM-1.8 stimulates macrophages into a pro-inflammatory phenotype: evaluation at transcriptional level. (a) Quantitative reverse transcription PCR (RT-qPCR) analysis of the levels of pro-inflammatory cytokines – tumor necrosis factor-α (TNF-α) and interleukin-12 (IL-12 p40) and (b) anti-inflammatory cytokines – transforming growth factor-β1 (TGF-β1) and interleukin-10 (IL-10), secreted by primary murine bone marrow-derived macrophages (BMDM) after 24 h of stimulation by acGM samples (* $P<0.05$ versus the phosphate buffer saline [PBS] group; ns: no significance; n=3). (c) Flow cytometry analysis of CD163 in BMDM after stimulation with acGM-1.8 (20, 50, and 100 μg/mL; * $P<0.05$ versus the BMDM-M0 group; ns: no significance; n=3). Data are representative for three independent experiments.

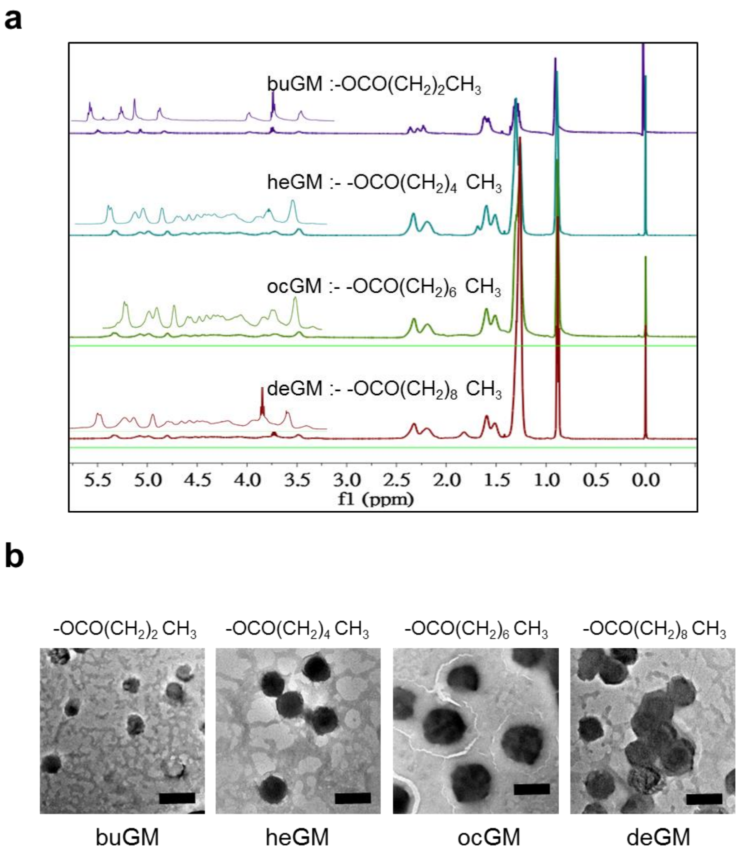

Supplementary Figure 3. Characterization of various GM derivatives. (a) ^{1}H NMR spectra. (b) Images of transmission electron microscopy (TEM) of four derivatives (buGM: $-OCO(CH2)_2CH_2$, heGM:$-OCO(CH2)_4CH_2$, ocGM: $-OCO(CH2)_6CH_2$, deGM: $-OCO(CH2)_8CH_2$, scale bar: 500 nm).

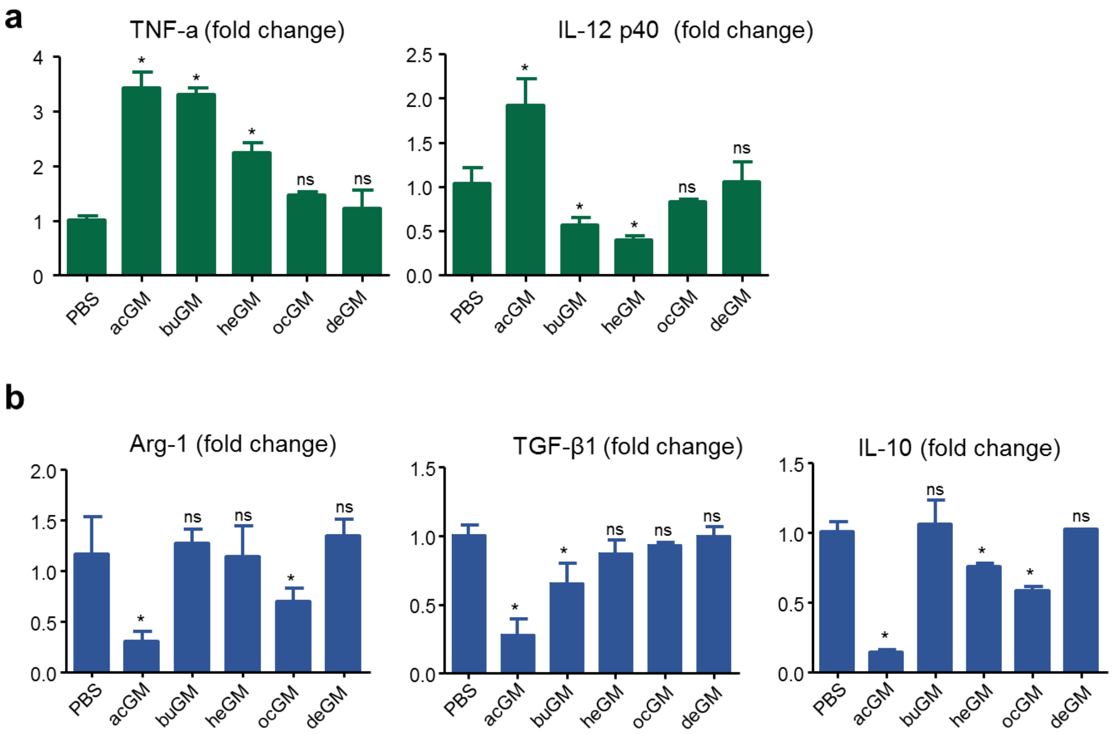

Supplementary Figure 4. Evaluation of the effect of various GM derivatives on macrophage phenotype: RT-qPCR analysis of the levels of (a) pro-inflammatory cytokines TNF-α and IL-12 p40 and (b) anti-inflammatory cytokines Arg-1, TGF-β1 and IL-10 by RT-qPCR, secreted by BMDM after 24 h of stimulation by various GM derivatives (* $P<0.05$ versus the PBS group; ns: no significance; n=3). Data are representative for three independent experiments.

a

	Acetyl group (%)	DS
acGM-1.8	24.04	1.81
acDex-1.9	33.96	1.91

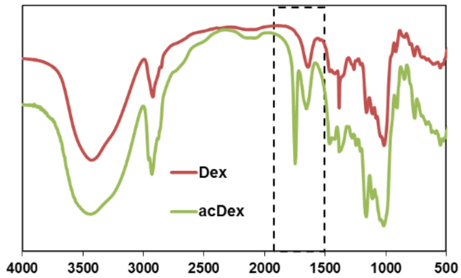

b

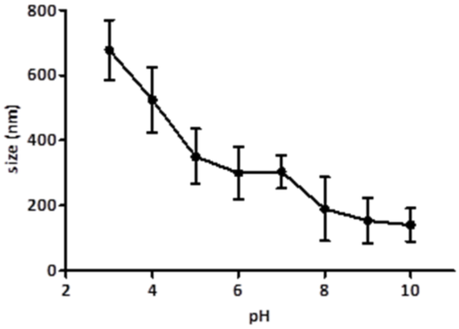

Supplementary Figure 5. (a) Characterization of acetylate dextran (acDex-1.9) by IR spectrum. (b) Measurement of particle size of acGM-1.8 in PBS with various pH (3-10), determined by dynamic light scattering (DLS); n=3; data are representative for three independent measurements.

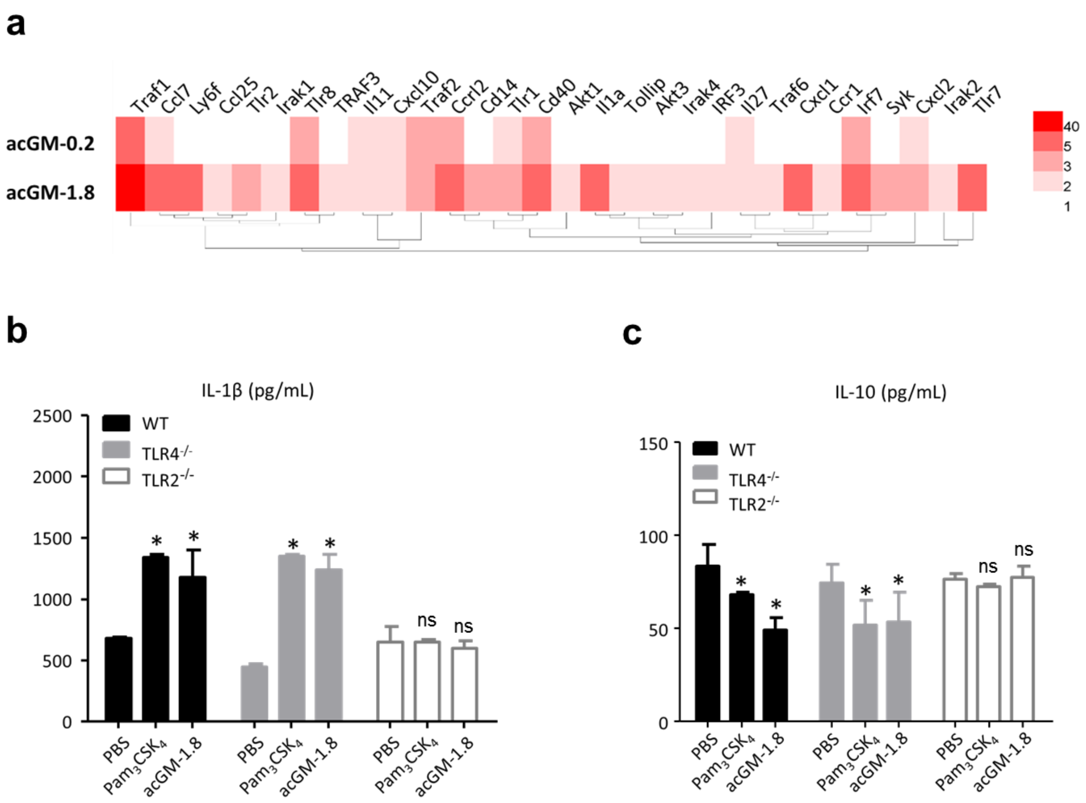

Supplementary Figure 6. (a) Heat map of gene expression related to toll-like receptor signaling pathway; the fold changes was defined according to color gradation. (b and c) Determination of cytokines (b) IL-1β and (c) IL-10 secreted by macrophages of TLR2/4$^{-/-}$ mice or wild type mice after the treatment of PBS, Pam_3CSK_4 or acGM-1.8 for 24h (* P<0.05 versus the PBS group; ns: no significance; n=3). Data are representative for three independent measurements.

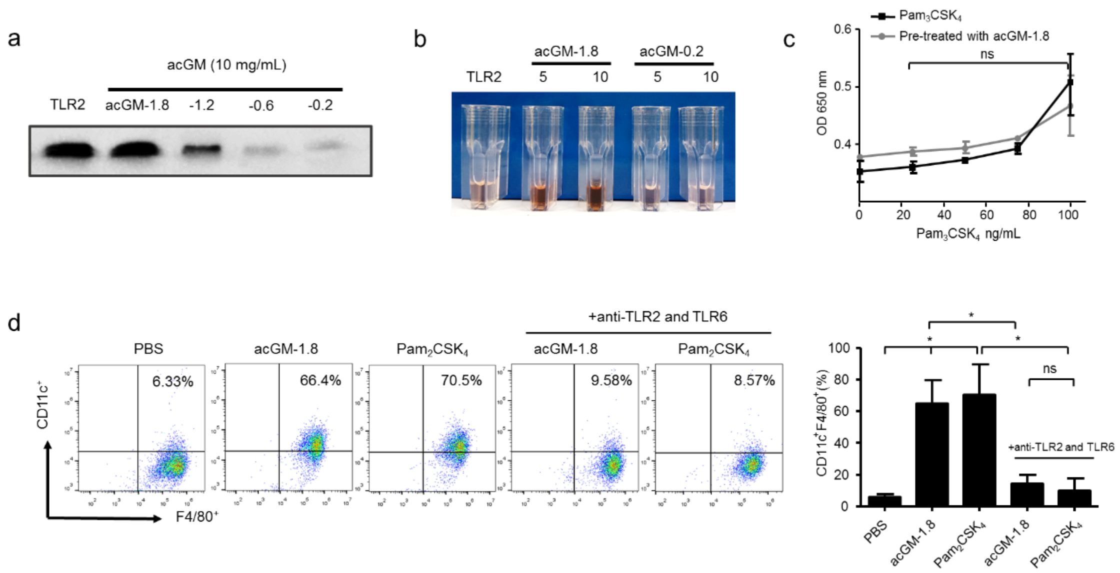

Supplementary Figure 7. Direct binding analysis between TLR2 and acGM-1.8, (a) acGM-1.8, -1.2, -0.6, or -0.2 (10 mg/mL) was co-incubated with the membrane proteins isolated from the lysate of TLR2 reporter cells at 4 °C overnight and the proteins were eluted for Western blotting. (b) Co-immunoprecipitation of TLR2 protein using acGM-1.8/0.2 (5 or 10 mg/mL) and detection of the polysaccharides by sulfuric-phenol. (c) Assessment of potential competition between acGM-1.8 and the classical TLR2 agonist Pam_3CSK_4: the TLR2 HEK-Blue cells were treated with Pam_3CSK_4 alone (1 to 100 ng/mL) or pre-treated with acGM-1.8 (1 µg/mL; 30 min) before Pam_3CSK_4 (1 to 100 ng/mL) was added, the level of secreted embryonic alkaline phosphatase (SEAP) was recorded by reading the absorbance at 650 nm. (* $P<0.05$ versus the PBS group; ns: no significance; n=3.) (d) Flow cytometry analysis of $CD11c^+$ variation after treated with acGM-1.8 (100 µg/mL) and Pam_2CSK_4 (100 ng/mL,TLR2/6 agonist) in BMDM with or without pre-treated with anti-TLR2 and anti-TLR6 (1µg/mL) for 24h, * $P<0.05$ between each two group; ns: no significance; n=3. Data are representative for three independent measurements.

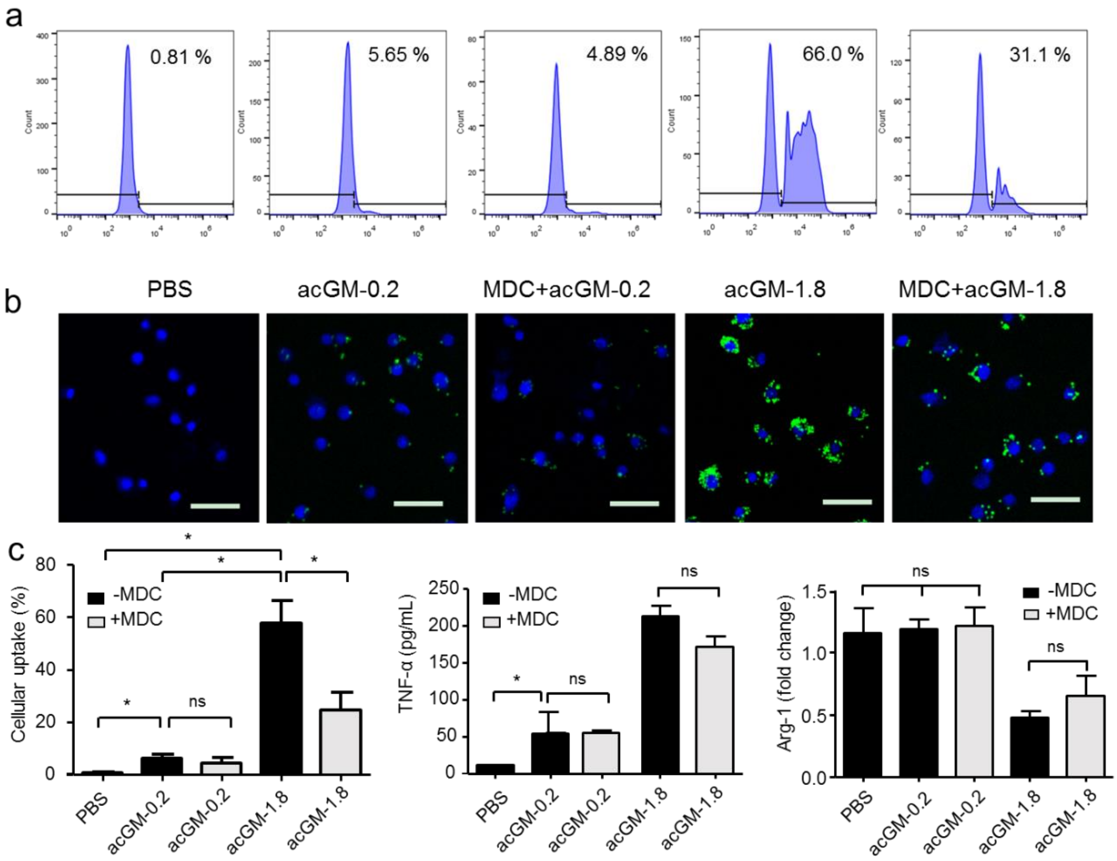

Supplementary Figure 8. Endocytosis did not play a key role in the acGM-1.8's function (a) Flow cytometry analysis of cellular uptake of FITC-labelled acGM-1.8/0.2 with or without pre-treated with Monodansylcadaverine (MDC, a clathrin inhibitor) for 0.5 h. (b) Confocal image of intracellular of FITC-labelled acGM-1.8/0.2 (Green), nucleus were stained with DAPI (Blue) (scale bar: 50 μm). (c) Statistical analysis of FITC-labelled acGM-1.8/0.2 cellular uptake in BMDM by flow cytometry analysis, * *P<0.05* between each two group; ns: no significance; n=3. (d) Measurement of TNF-a by ELISA (left) and Fold change of Arg-1 expression by RT-qPCR (right) with or without pre-treated with MDC, * *P<0.05* between each two group; ns: no significance; n=3. Data are representative for three independent measurements.

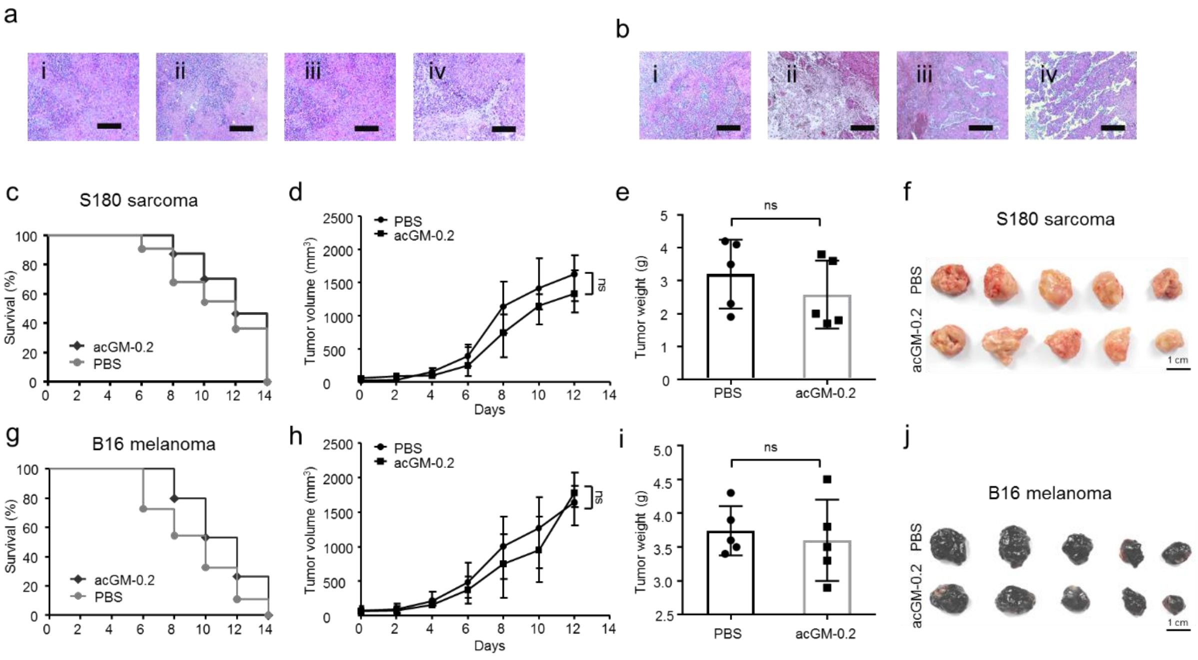

Supplementary Figure 9. (a and b) H&E staining image of the treatment procedure of acGM-1.8 in two types of tumors, S180 sarcoma and B16 melanoma, growing in mice. When the tumor diameter reached 0.5 cm, the mice were randomly divided into four groups and treated with: i) PBS for 14 days; ii) acGM-1.8 (10 mg/kg) for 14 days; iii) acGM-1.8 (10 mg/kg) for 14 days and then PBS for 14 days; iv) acGM-1.8 for 28 days. PBS or acGM-1.8 was intratumorally injected every two days. (c-j) Assessment of antitumor effect of acGM-0.2. (c) Survival ratio of the S180 sarcoma-bearing mice in PBS and acGM-0.2 groups. (d) Measurement of the S180 tumor size in PBS and acGM-0.2 groups; * *P<0.05* versus the PBS group, ns: no significance; n=5. (e) Measurement of the S180 tumor weight in PBS and acGM-0.2 groups; * *P<0.05* versus the PBS group, ns: no significance; n=5. (f) Gross view of the S180 tumor samples in PBS and acGM-0.2 groups; n=5. (g) Survival ratio of the B16 melanoma-bearing mice in PBS and acGM-0.2 groups. (h) Measurement of the B16 melanoma tumor size in PBS and acGM-0.2 groups; * *P<0.05* versus the PBS group, ns: no significance; n=5. (i) Measurement of the B16 melanoma tumor weight in PBS and acGM-0.2 groups; * *P<0.05* versus the PBS group, ns: no significance; n=5. (j) Gross view of the B16 tumor samplest in PBS and acGM-0.2 groups, n=5.

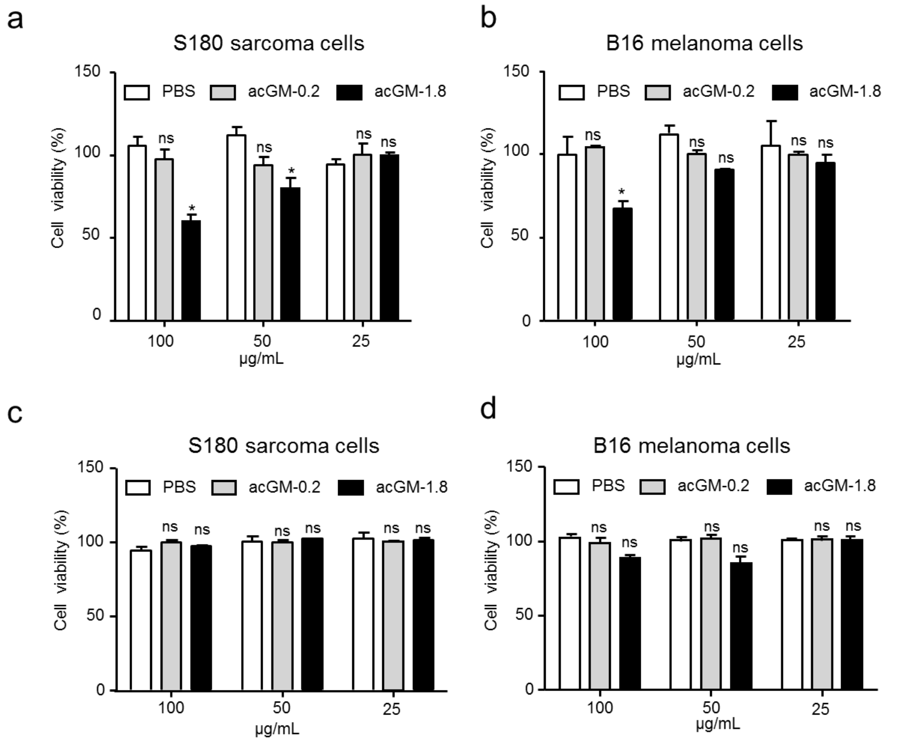

Supplementary Figure 10. Evaluation of acGM-1.8 antitumor effect *In vitro* of S180 sarcoma and B16 melanoma. (a and b) Cell viability tested by CCK-8 assay of S18 sarcoma cells (a) and B16 melanoma cells (b) after co-incubated with collected culture medium of BMDMs after stimulated by acGM-1.8/0.2 for 24 h. (c and d) Cell viability tested by CCK-8 assay of S18 sarcoma cells (c) and B16 melanoma cells (d) after directly treated with acGM-1.8/0.2. (* $P<0.05$ versus the PBS group; ns: no significance; n=3) Data are representative for three independent measurements.

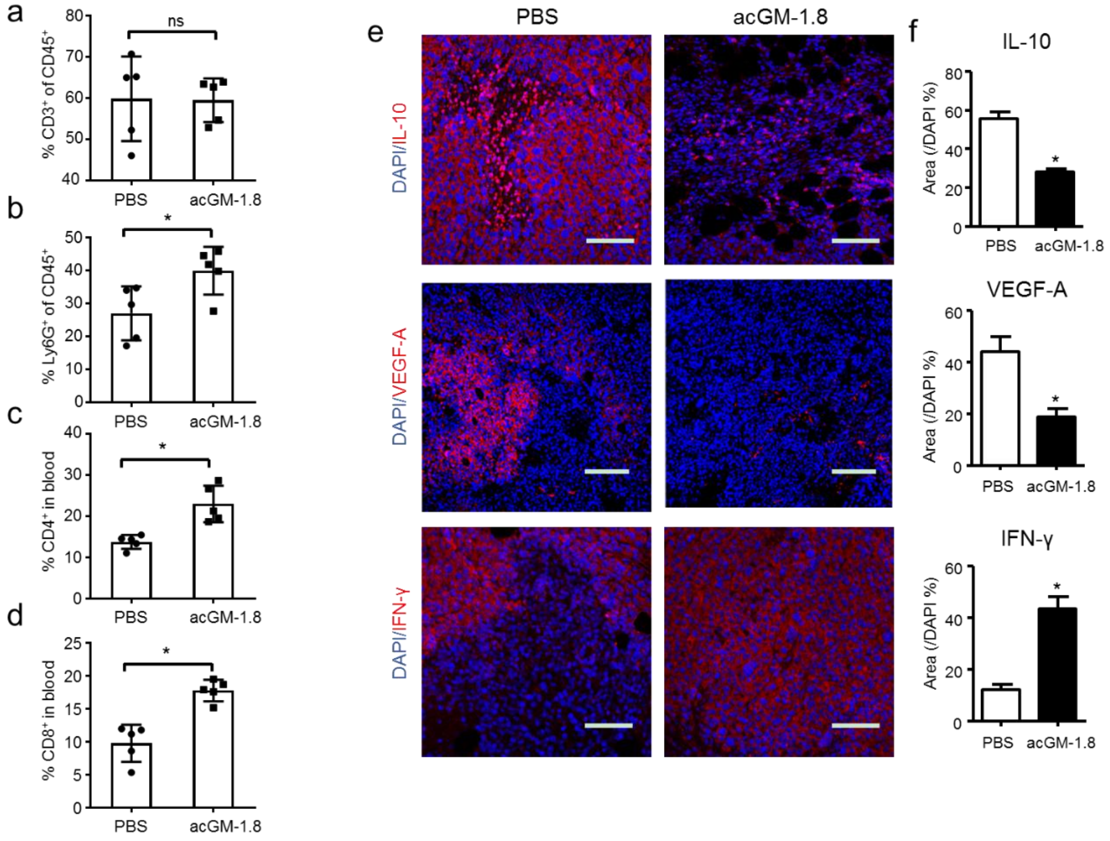

Supplementary Figure 11. S180-bearing mice after 14 days acGM-1.8 or PBS treatment, tumor tissue was harvested, smashed and digested, before analyzed with flow cytometry. (a) Proportion of T lymphocyte $CD3^{+}$of $CD45^{+}$ positive cells. (b) Proportion of neutrophils $Ly6G^{+}$of $CD45^{+}$ positive cells. * *P<0.05* versus the PBS group; ns: no significance; n=5.(c and d) Proportion of $CD4^{+}$ (c) and $CD8^{+}$ (d) T cells of the blood in S180-bearing mice after treated with acGM-1.8 or PBS for 14 days, peripheral-blood was collected by removing eyeballs from the mice and analysed by flow cytometry. * *P<0.05* versus the PBS group; ns: no significance; n=5. (e) Immunofluorescence staining of IL-10, VEGF-A and IFN-γ (red) in tumor tissue after acGM-1.8 treatment (nuclear was stained with Blue-DAPI, scale bar: 100 μm). (f) Quantitative analysis of the immunofluorescent images of IL-10, VEGF-A and IFN-γ. * *P<0.05* versus the PBS group; ns: no significance; n=5. For each protein, the quantification was performed based on samples from three mice (five areas per each mouse).

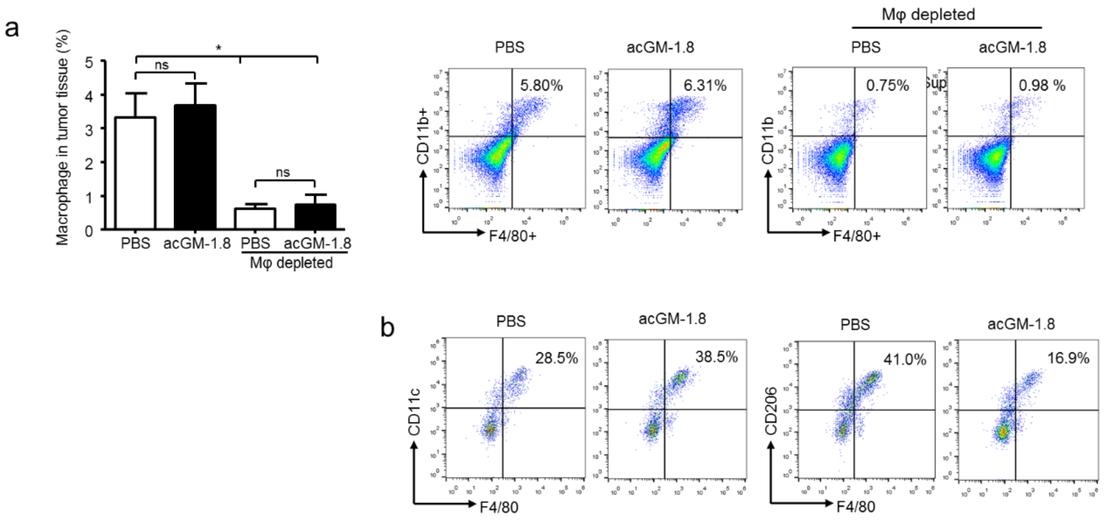

Supplementary Figure 12. (a) Depletion macrophages *in situ* in S180 tumor-bearing mice by clodronate liposomes, liposomes (5 mg/mL, 50 μL) was pre-intratumorally injected for 24 h followed by the injection of PBS and acGM-1.8. After the 14-day treatment, tumour tissues were collected, smashed and digested to single cells. The proportion of macrophage in tumour was stained with F4/80 and CD11b and analysed by flow cytometry. * $P<0.05$ between each two group; ns: no significance; n=5. (b) Corresponding flow cytometry analysis of proportion change of M1/M2-type macrophages of CD45+ positive cells in tumor tissue of nude mice after intratumorally injected with PBS and acGM-1.8, * $P<0.05$ versus PBS treatment; ns: no significance; n=5.

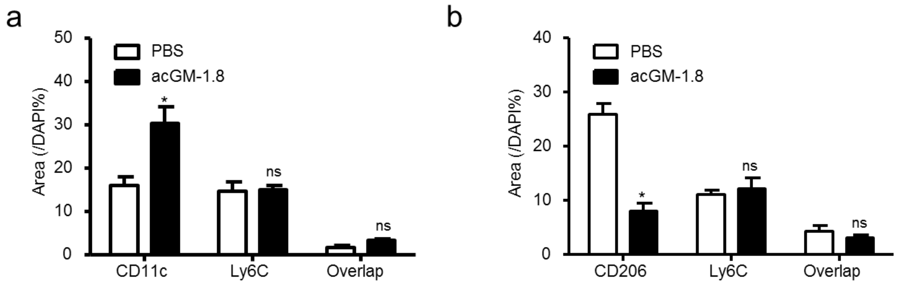

Supplementary Figure 13. Quantitative analysis of the immunofluorescent images of co-staining with Ly6C^{+} and CD11c^{+} (a) and co-staining with Ly6C^{+} and CD206^{+} (b) in tumour tissue, * *P<0.05* versus PBS treatment; ns: no significance; n=5.

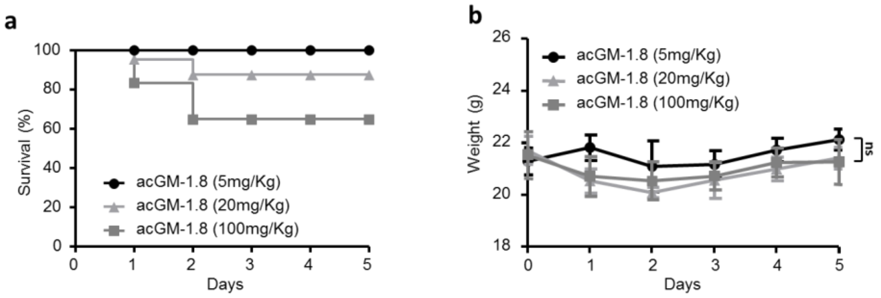

Supplementary Figure 14. Survival rate and animal weight after the treatment of TLR agonists. (a) Survival rate after intraperitoneal injection of acGM-1.8 with various doses (5,20, 100 mg/Kg with mouse) (b) Measurement of the animal weight after intraperitoneal injection of acGM-1.8 with various doses (5, 20, 100 mg/Kg with mouse,) ns: no significance.

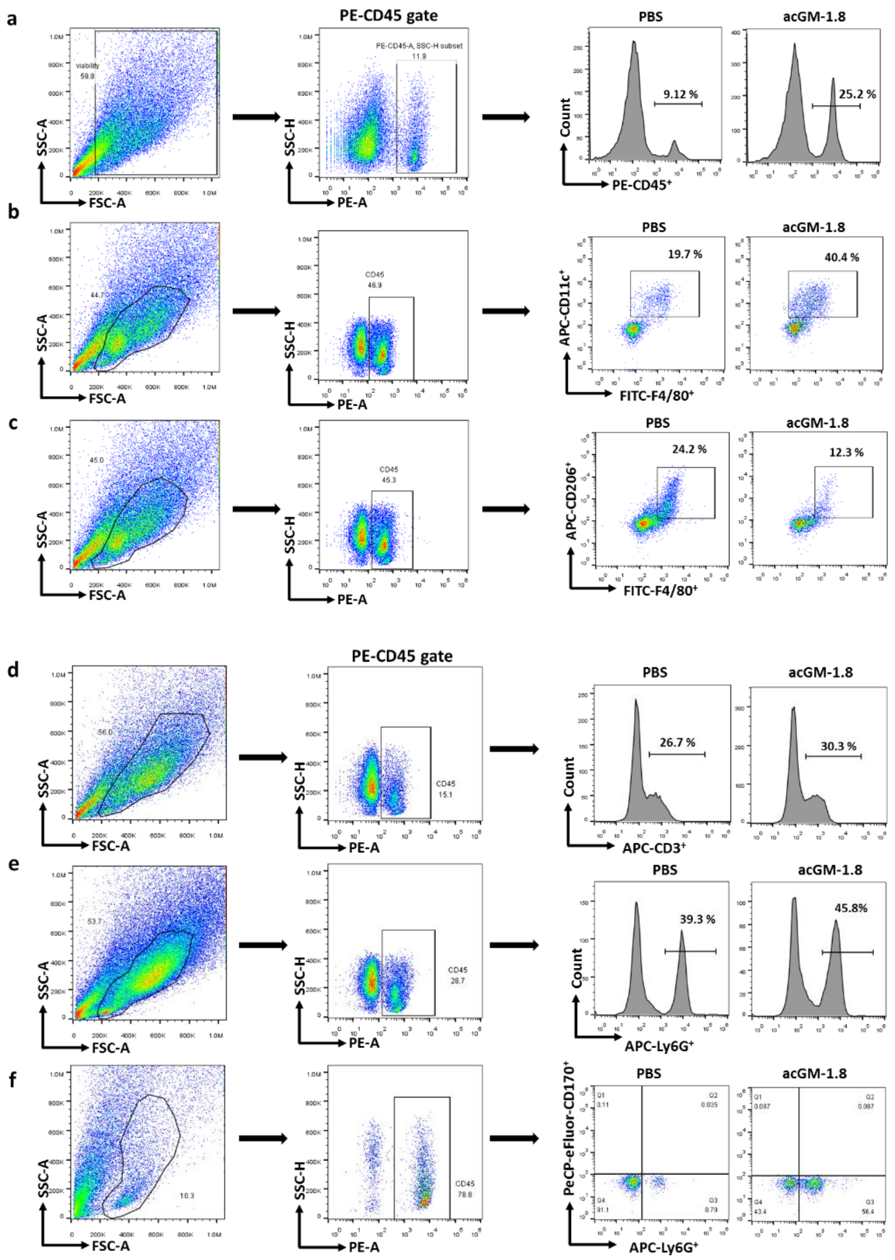
a
b
c
d
e
f
PE-CD45 gate
PBS
acGM-1.8
9.12 %
25.2 %
19.7 %
40.4 %
24.2 %
12.3 %
26.7 %
30.3 %
39.3 %
45.8%
SSC-A
FSC-A
SSC-H
PE-A
Count
PE-CD45+
APC-CD11c+
FITC-F4/80+
APC-CD206+
APC-CD3+
APC-Ly6G+
PeCP-eFluor-CD170+

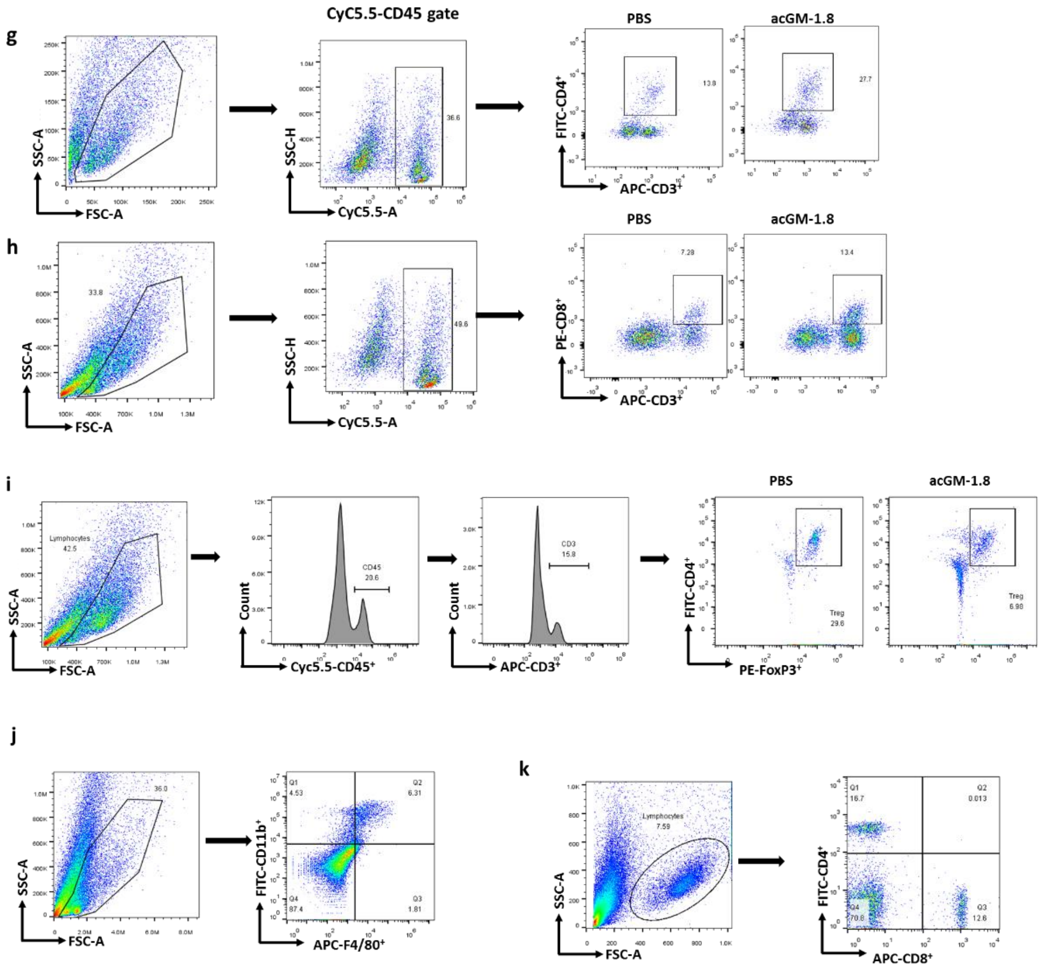

Supplementary Figure 15. Gating strategies used for cell sorting. (a) $CD45^+$positive cell in tumour tissue; (b) M1-type macrophages macrophage F4/80^+ $CD11c^+$; (c) M2-type macrophages macrophage F4/80^+ $CD206^+$; (d) $CD3^+$ positive cell of $CD45^+$ leukocyte cells; (e) $Ly6G^+$ positive cell of $CD45^+$; (f) Eosnophils cells $CD170^+Ly6G^+$ of $CD45^+$ leukocyte cells; (g) Regulatory T cells $CD4^+$ $Foxp3^+$ of $CD45^+$ positive cells; (h) T lymphocyte $CD4^+$ of $CD45^+$ positive cells and (i) T lymphocyte $CD8^+$ of $CD45^+$ positive cells used in the *in vivo* antitumor assessment of acGM-1.8 presented in Fig. 6a-f and Supplementary Fig. 11a and b. (j) Macrophage F4/80^+ $CD11b^+$ used for the depletion of macrophages in S180 sarcoma-bearing mice for validation in Fig. 6h; (k) $CD4^+$ T lymphocyte and $CD8^+$ T lymphocyte in the blood used in the *in vivo* antitumor assessment of acGM-1.8 presented in Supplementary Fig. 11c and d.

Supplementary References

1. Feng Y*, et al.* A macrophage-activating, injectable hydrogel to sequester endogenous growth factors for in situ angiogenesis. *Biomaterials* **134**, 128-142 (2017).
