# Supplementary material for: A toll-like receptor agonist mimicking microbial signal to generate tumor-suppressive macrophages
Source: Nat Commun. 2019 May 22;10:2272. doi: 10.1038/s41467-019-10354-2 (PMC6531447; doi:10.1038/s41467-019-10354-2)
Supplement: Supplementary file 3 — Reporting Summary [file 41467_2019_10354_MOESM3_ESM.pdf]

nature research

Corresponding author(s): NCOMMS-18-34327A, Chunming Wang

Reporting Summary

Nature Research wishes to improve the reproducibility of the work that we publish. This form provides structure for consistency and transparency in reporting. For further information on Nature Research policies, see Authors & Referees and the Editorial Policy Checklist.

Statistical parameters

When statistical analyses are reported, confirm that the following items are present in the relevant location (e.g. figure legend, table legend, main text, or Methods section).

n/a	Confirmed	
☐	☒	The exact sample size (n) for each experimental group/condition, given as a discrete number and unit of measurement
☐	☒	An indication of whether measurements were taken from distinct samples or whether the same sample was measured repeatedly
☐	☒	The statistical test(s) used AND whether they are one- or two-sided<br>*Only common tests should be described solely by name; describe more complex techniques in the Methods section.*
☒	☐	A description of all covariates tested
☐	☒	A description of any assumptions or corrections, such as tests of normality and adjustment for multiple comparisons
☐	☒	A full description of the statistics including central tendency (e.g. means) or other basic estimates (e.g. regression coefficient) AND variation (e.g. standard deviation) or associated estimates of uncertainty (e.g. confidence intervals)
☐	☒	For null hypothesis testing, the test statistic (e.g. F, t, r) with confidence intervals, effect sizes, degrees of freedom and P value noted<br>*Give P values as exact values whenever suitable.*
☒	☐	For Bayesian analysis, information on the choice of priors and Markov chain Monte Carlo settings
☒	☐	For hierarchical and complex designs, identification of the appropriate level for tests and full reporting of outcomes
☒	☐	Estimates of effect sizes (e.g. Cohen's d, Pearson's r), indicating how they were calculated
☐	☒	Clearly defined error bars<br>*State explicitly what error bars represent (e.g. SD, SE, CI)*

Our web collection on statistics for biologists may be useful.

Software and code

Policy information about availability of computer code

Data collection	1.TEM images were captured using transmission electron microscopy JEOL 2100F (200KV.0.14nm) JEOL Ltd.<br>2.Flow cytometry data were acquired using BD Accuri C6.<br>3.Haematoxylin and eosin (H&E) staining were captured using Axio Imager A2 microscope<br>4.All immunostaining pictures were captured using a Leical TCS SP8 confocal microscope<br>5.NMR data was acquired using BZH 600MHz/54 mm ASCEND, BRUKER Ltd.<br>6.Quantitative real-time PCR data were collected using Agilent Technologies Stratagene Mx3005P<br>7.Mircoarry data was acquired by Agilent Microarray Scanner (Cat.# G2565CA, Agilent technologies, Santa Clara, CA, US)<br>8. Particle size was collected using Zetasizer Nano ZS, Malvern Panalytical Ltd.<br>9. UV data was collected using FlexStation III Multi-Mode Microplate Reader
Data analysis	1.Flow cytometry data were analyzed using FlowJo X 0.7.<br>2.Mircoarry data were extracted with Feature Extraction software 10.7 (Agilent technologies, Santa Clara, CA, US). Raw data were normalized by Quantile algorithm, KEGG Pathway analysis were performed in the standard enrichment computation method according to the KEGG database (https://www.genome.jp/kegg).<br>3.Statistical analysis was performed using Graphpad Prism 7.01.

For manuscripts utilizing custom algorithms or software that are central to the research but not yet described in published literature, software must be made available to editors/reviewers upon request. We strongly encourage code deposition in a community repository (e.g. GitHub). See the Nature Research guidelines for submitting code & software for further information.

Data

Policy information about availability of data

All manuscripts must include a data availability statement. This statement should provide the following information, where applicable:
- Accession codes, unique identifiers, or web links for publicly available datasets
- A list of figures that have associated raw data
- A description of any restrictions on data availability

All relevant data that are included with this study are available from corresponding author upon reasonable request. The source data underlying Figure 2b-g, 3c-e and h, 4d-k, 5b, d, e, f, h and i, 6a-l and o, Figure 7, and Supplementary Figures 2, 4, 6b and c, 7b and d, 8, 9, 10, 13 and 14 are provided as a Source Data file

Field-specific reporting

Please select the best fit for your research. If you are not sure, read the appropriate sections before making your selection.

☒ Life sciences ☐ Behavioural & social sciences ☐ Ecological, evolutionary & environmental sciences

For a reference copy of the document with all sections, see nature.com/authors/policies/ReportingSummary-flat.pdf

Life sciences study design

All studies must disclose on these points even when the disclosure is negative.

Sample size	Sample size was determined empirically for sufficient statistical power. Variations between samples were also used to determine the suitability of the sample size.
Data exclusions	No data were excluded from analyses in the experiments.
Replication	All attempts at replication were successful.
Randomization	In animal studies, we randomly allocated animals into groups such that as animals were added to the experiment, the numbers of animals in each group did not significantly differ.
Blinding	We needed to investigate the difference between different groups, and such difference had not been known. Thus we did not use blinding in the study.

Reporting for specific materials, systems and methods

Materials & experimental systems

n/a	Involved in the study	
☒	☐	Unique biological materials
☐	☒	Antibodies
☐	☒	Eukaryotic cell lines
☒	☐	Palaeontology
☐	☒	Animals and other organisms
☒	☐	Human research participants

Methods

n/a	Involved in the study	
☒	☐	ChIP-seq
☐	☒	Flow cytometry
☒	☐	MRI-based neuroimaging

Antibodies

Antibodies used

1. PE anti-mouse CD11C antibody,BD Biosciences,cat. #:557401
2. APC anti-mouse F4/80 antibody,Biolegend, cat. #: 123115
3. FITC anti-mouse CD206 antibody,Biolegend, cat. #:141703
4. FITC anti-mouse CD4 antibody, Biolegend, cat. #:100510
5. APC anti-mouse CD8a antibody, Biolegend, cat. #:100712
6. Anti-F4/80 antibody (Rabbit monoclonal to F4/80), abcam, cat. #: ab100790
7. Anti-CD11c antibody (Mouse monoclonal to CD11c), abcam,cat. #:ab11029
8. Anti-IL10 antibody (Rabbit polyclonal to IL-10), Cat. #: ab9969
9. Anti-interferon-gamma antibody (Rabbit monoclonal to interferon gamma), abcam,cat. #: ab133566
10. Anti-VEGF antibody (Rabbit monoclonal to VEGFA), abcam,cat. #: 52917

	11. Anti-TLR2 antibody (Mouse monoclonal to TLR2), abcam, cat.#:ab24192<br>12.PE-CD45 antibody , Biolegend,cat. #:103105<br>13.Cyc5.5-CD45 antibody, Biolegend,cat. #:103131<br>14.APC-CD3 antibody, Biolegend,cat. #:100235<br>15.FITC-CD4 antibody, Biolegendcat. #:100510<br>16.APC-CD8a antibody, Biolegend cat. #:100712<br>17.PE-FoxP3 antibody, Biolegend cat. #:126403<br>18.APC-Ly6G antibody, Biolegend cat. #:127613<br>19.PercP-eFluor-CD170 antibody, Biolegend cat. #:46-1702-82<br>20.FITC-anti-mouse Ly6C abcam, cat.#:ab15686<br>21.anti-CD11 antibody abcam, cat.#:ab11029<br>22. anti-CD206 antibody abcam, cat.#:ab64693
Validation	Antibody 1-5 were purchased for flow cytometry. All the antibodies are widely published and have quality control (QC) tested per the company's standard procedure.<br>1. PE anti-mouse CD11C antibody was purchased from BD Biosciences for flow cytometry, citation:J Immunol. 2003; 170(12):5927-5935.<br>2.APC anti-mouse F4/80 antibody citation: Ruedl C, et al. 1996. Eur. J. Immunol. 26:1801<br>3.FITC anti-mouse CD206 antibody citation:Sun L, et al. 2015. J Immunol. 194:4891<br>4. FITC anti-mouse CD4 antibody, citation:J Immunol. 2007 Feb;178(4):2499-506.<br>5. APC anti-mouse CD8a antibody, citation: Int Immunol. 2005 Dec;17(12):1607-17. Epub 2005 Nov 1.<br>Antibody 6-10 were purchased for immunofluorescence staining.Application-specific criteria was used to pass or fail the antibodies, and all of antibodies were typically tested in multiple applications (has to show specific signal for the primary application, needs to be tested on positive and negative control samples where available and any potential cross-reaction has to be noted and supported with data from sequence alignment, internal testing, and/or external publications), please refer to the the manufacturer's description: http://www.abcam.com/primary-antibodies/how-we-validate-our-antibodies.<br>6.Anti-F4/80 antibody citation:Zhang H et al. Nat Commun 8:15016 (2017).<br>7.Anti-CD11c antibody citation: Kimbrough D et al. J Mol Cell Cardiol 119:51-63 (2018).<br>8. Anti-IL10 antibody citation:Xu Y et al. J Immunol 196:2476-2491 (2016).<br>9. Anti-interferon-gamma antibody citation:Dufour A et al. Nat Commun 9:2416 (2018)<br>10. Anti-VEGF antibody citation:Hara T et al. Proc Natl Acad Sci U S A 114:E4416-E4424 (2017)<br>Antibody 11 was used for western blotting. and co-immunoprecipitation.<br>11 Anti-TLR2 antibody citation:Flo TH et al. J Immunol 164:2064-9 (2000)<br>12-19.Antibodies were used for flow cytometry analysis of immure cells in tumor<br>20-22. Antibodies were used for immunofluorescence staining.

Eukaryotic cell lines

Policy information about cell lines

Cell line source(s)	1.Mouse sarcoma cell line S180 cells obtained from Institute of Biochemistry and Cell Biology.<br>2. B16-F10 melanoma cell line obtained from Institute of Biochemistry and Cell Biology.<br>3.Murine TLR2-expressing HEK 293 cells were purchased from InvivoGen Ltd.<br>4.Murine TLR4-expressing HEK 293 cells was purchased from InvivoGen Ltd.
Authentication	1&2 Cell lines were authenticated by Beijing Microread Genetics Co., Ltd using STR analysis. More information is provided in Report of Human Cell Line Authentication.<br>3. According to the manufacturer's description: Murine TLR2-expressing HEK 293 cells have been stimulated by various pathogen recognition receptor (PRR) agonists. As expected, TLR2 agonists induced the production of SEAP. The expression of murine TLR2 in this cell line has been validated using fluorescence-activated cell sorting (FACS). The expression of the murine TLR2 and CD14 genes has been confirmed by RT-PCR. The stability of this cell line for 20 passages following thawing has been verified.<br>4. According to the manufacturer's description: Murine TLR4-expressing HEK 293 cells have been stimulated by various pathogen recognition receptor (PRR) agonists. As expected, TLR4 agonists induced the production of SEAP.Expression of mouse TLR4 and MD-2/CD14 genes has been confirmed by RT-PCR. The cell surface expression of mouse TLR4 in this cell line has been validated using fluorescence-activated cell sorting (FACS). The stablity of this cell line for 20 passages following thawing has been verified.
Mycoplasma contamination	1&2 Cells were confirmed negative for mycoplasma contamination. Mycoplasma Detection Authentication was performed by Beijing Microread Genetics Co., Ltd using Mycoplasma Detection Set Kit.) More information is provided in Report of Mycoplasma Detection Authentication.<br>3&4 Cell lines were guaranteed mycoplasma-free by the manufacturer.
Commonly misidentified lines<br>(See ICLAC register)	No commonly misidentified cell lines were used.

Animals and other organisms

Policy information about studies involving animals; ARRIVE guidelines recommended for reporting animal research

Laboratory animals	C57BL/6 mice were purchased from Vital River Laboratories (Beijing, China).<br>TLR4 KO mice (Tlr4lps-del, C57BL/10ScN background) and TLR2 KO mice (B6.129-Tlr2tmlKir/J, C57BL/6J background) and nude

mice were purchased from Jackson Laboratory (USA) through Nanjing Biomedical Research Institute of Nanjing University (China)

Wild animals

n/a

Field-collected samples

The animals were fed in a specific pathogen-free (SPF) animal facility with controlled light (12 h light/dark), temperature and humidity, with food and water available. Animal protocols were reviewed and approved by the Animal Care and Use Committee of Nanjing University, and conformed to the Guidelines for the Care and Use of Laboratory Animals published by the National Institutes of Health.

Flow Cytometry

Plots

Confirm that:

☒ The axis labels state the marker and fluorochrome used (e.g. CD4-FITC).

☒ The axis scales are clearly visible. Include numbers along axes only for bottom left plot of group (a 'group' is an analysis of identical markers).

☒ All plots are contour plots with outliers or pseudocolor plots.

☒ A numerical value for number of cells or percentage (with statistics) is provided.

Methodology

Sample preparation

To analyze blood samples, the whole blood was collected in K2EDTA collection tubes (Terumo Medical, Somerset, NJ, USA)and treated with red blood cell lysis buffer. To analyze adherent cells (Bone marrow derived macrophages), cells was washed with PBS and collected with scraper. All kinds of cells were adjusted to 10^5 cells/mL and blocked with 2% bovine serum albumin (BSA) and incubated with the fluorescence-conjugated antibodies in the dark for 30 min at 4 °C according to product manuals.

Instrument

BD Accuri C6, The CytoFLEX Platform

Software

Flowjo software (Tree Star, San Diego, CA, USA)

Cell population abundance

n/a

Gating strategy

All samples were stained with antibodies or isotype control. All samples were gated on FSC-A/SSC-A to remove debris and then FITC-H, PE-H, APC-H and CyC5.5-H were used to differentiate positive or negative populations. Cells stained with isotype control was used to define negative population.

☒ Tick this box to confirm that a figure exemplifying the gating strategy is provided in the Supplementary Information.
